# Stress-Free Intubation With Preserved Spontaneous Breathing for Cases With Difficult Airway Management: A Case Series and Retrospective Analysis

**DOI:** 10.7759/cureus.89393

**Published:** 2025-08-05

**Authors:** Yuki Kojima, Shinya Endo, Kazuya Hirabayashi

**Affiliations:** 1 Anesthesiology, Asahi General Hospital, Asahi, JPN

**Keywords:** analgesia, anesthesia, awake intubation, intratracheal intubation, ultrasound-guided nerve block

## Abstract

Awake tracheal intubation (ATI) is a crucial technique for difficult airway management, particularly in patients with obesity, restricted neck movement, or upper airway abnormalities. Despite its efficacy, ATI is often avoided because of the technical challenges and stress it imposes on patients and anesthesiologists. We describe a new method, termed "intubation maintaining spontaneous breathing with three nerve blocks technique" (3N technique), which leverages nerve blocks to suppress reflexes, preserve spontaneous breathing, and facilitate smooth intubation. The 3N technique requires minimal equipment and staff, thus reducing procedural stress and time. This new approach combines ultrasound-guided selective glossopharyngeal nerve block, superior laryngeal nerve block, and translaryngeal block. The data collected included the time from anesthesia initiation to intubation, types and dosages of sedatives and analgesics, patient discomfort during induction, and memory at induction. In our retrospective analysis of 18 cases, no patient experienced discomfort or remembered the procedure, and intubation was completed in an average of 16 minutes. Although the 3N technique is not suitable for patients with a full stomach or pediatric cases, it is broadly applicable and offers a safer and simpler alternative to conventional ATI methods, especially in patients with difficult airway management.

## Introduction

Awake tracheal intubation (ATI) is defined as the successful placement of a tracheal tube in a patient who is either awake or lightly to moderately sedated while maintaining spontaneous respiration [1-3]. The advantages of ATI include intubation with minimal administration of anesthetic agents and the preservation of protective respiratory reflexes until the procedure is completed. ATI is particularly recommended for patients with predicted difficulty in airway management or those who may not tolerate the apneic phase [4]. Additionally, ATI is indicated for patients requiring cervical spine immobilization or those with anatomical abnormalities in the upper airway [5]. Given its high success rates and favorable safety profile, ATI remains the gold standard for managing anticipated difficult airway scenarios such as "cannot ventilate, cannot intubate." Despite its advantages, ATI is used in only 0.2% of all tracheal intubations in the United Kingdom [6]. The disadvantages of ATI include the time required to perform it and the challenge of managing the experiences and movements of awake patients [7].

Recent advancements in nerve block techniques have proven effective in suppressing sensory and reflex responses in the pharynx and neck. A superior laryngeal nerve block provides sensory blockade to the vocal cords, abolishes the glottic closure reflex, and blunts the sensation of the structures above the cords [8-10], whereas a translaryngeal block targets the sensory nerves of the trachea below the vocal cords [10]. Furthermore, ultrasound-guided selective glossopharyngeal nerve block effectively suppresses the gagging reflex [11,12]. These techniques are promising adjuncts for facilitating ATI. Therefore, we introduce a new approach that employs these advanced nerve block techniques for difficult airway management (DAM) in this report. Additionally, we conducted a retrospective analysis of 18 cases using this method to assess patient stress levels, intubation duration, and complications.

## Materials and methods

Ethical approval and informed consent

Written informed consent was obtained from all patients for study inclusion and the publication of associated images and video. This study was approved by the Ethics Review Board of Asahi General Hospital (approval number: 2025012112) and was conducted in accordance with the principles outlined in the Declaration of Helsinki and its subsequent amendments.

Intubation maintaining spontaneous breathing with the three nerve blocks technique

**Figure 1:**
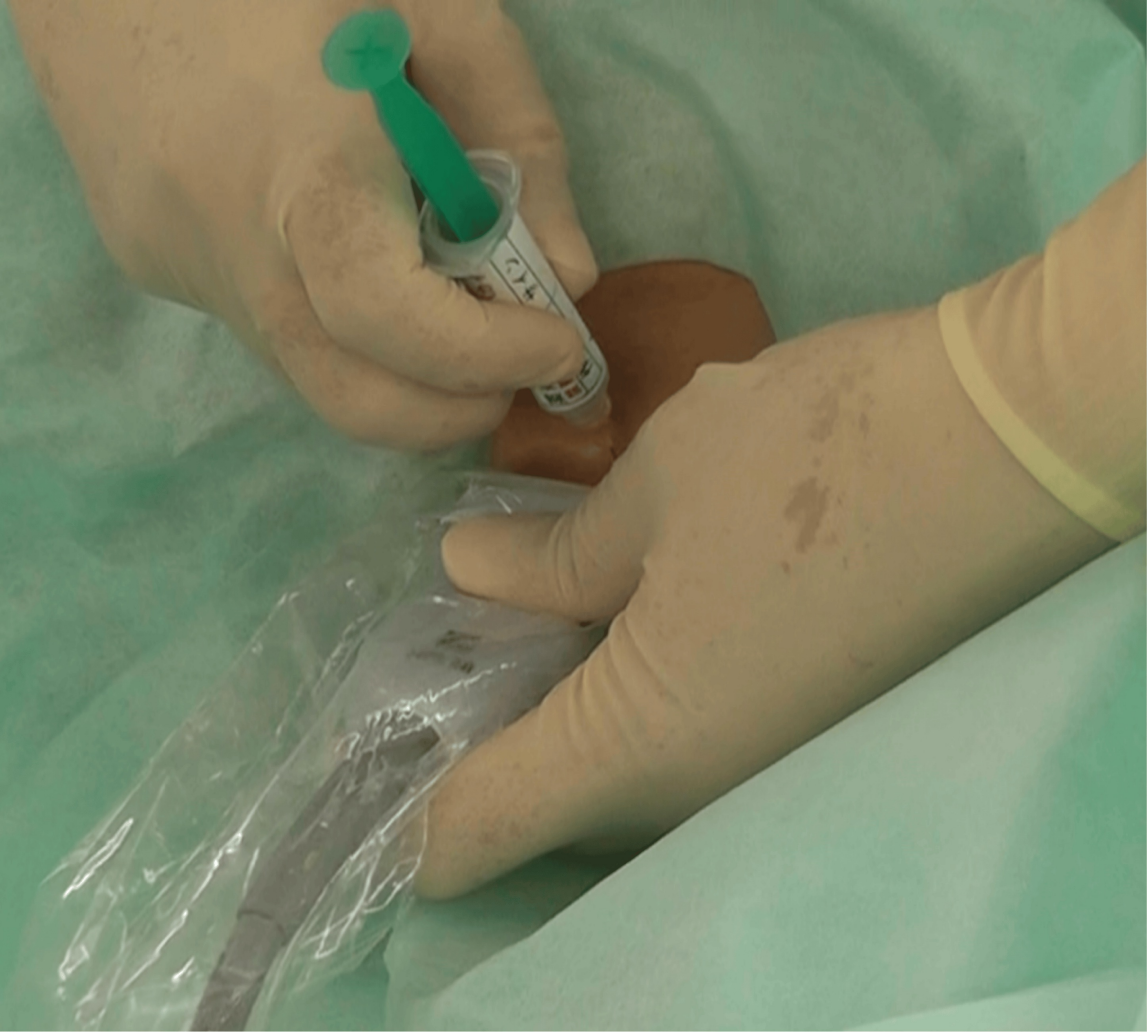
Ultrasound-guided selective glossopharyngeal nerve block to suppress the gagging reflex To perform intubation while maintaining spontaneous breathing with the three nerve blocks technique (3N technique), we first performed an ultrasound-guided selective glossopharyngeal nerve block. Reflex suppression was achieved after the block was performed.

This novel approach, termed the "3N technique," was performed using a linear ultrasound probe (SonoSite SII; Fujifilm, Tokyo, Japan). To perform the 3N technique, patients were placed in a supine position and administered intravenous midazolam to achieve a sedation score of 2-4 on the Ramsay scale or Verrill sign II [13,14]. For ultrasound-guided selective glossopharyngeal nerve block, the patient's head was first turned toward the anesthesiologist (Figure 1).

**Figure 2:**
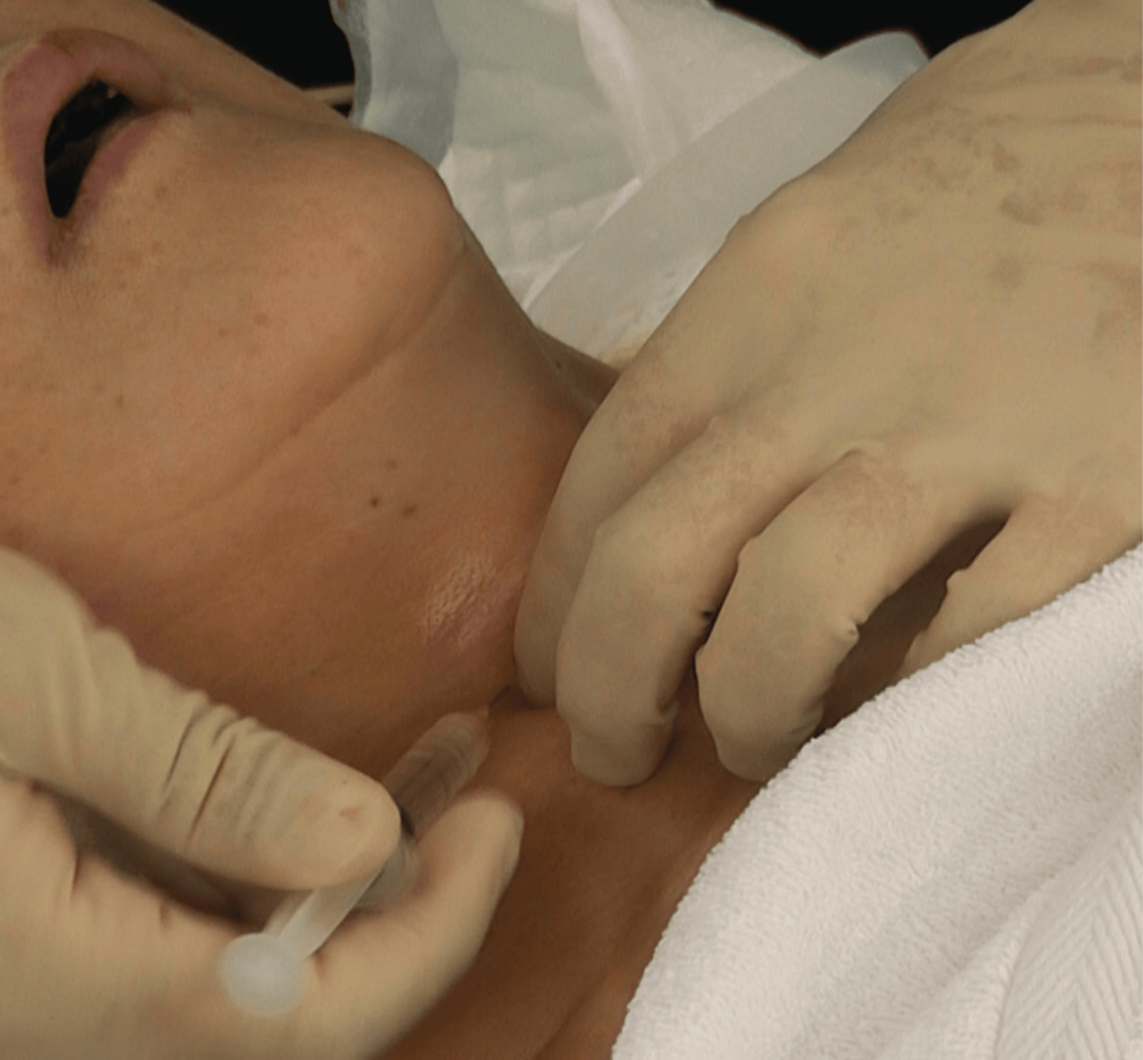
Superior laryngeal nerve block to control the glottic closure reflex and blunt the sensation of structures above the cords After ultrasound-guided selective glossopharyngeal nerve block, a bilateral superior laryngeal nerve block is performed. This nerve block is easier to perform when the neck is extended.

A linear ultrasound probe was then used to identify the sternocleidomastoid and stylohyoid muscles. Subsequently, a 25-gauge, 25-mm needle was inserted deep into the stylohyoid muscle using an out-of-plane approach, and 2 mL of 1% lidocaine was injected bilaterally to suppress the gagging reflex [11]. The patient's head was positioned forward for a superior laryngeal nerve block (Figure 2) [9,15].

**Figure 3:**
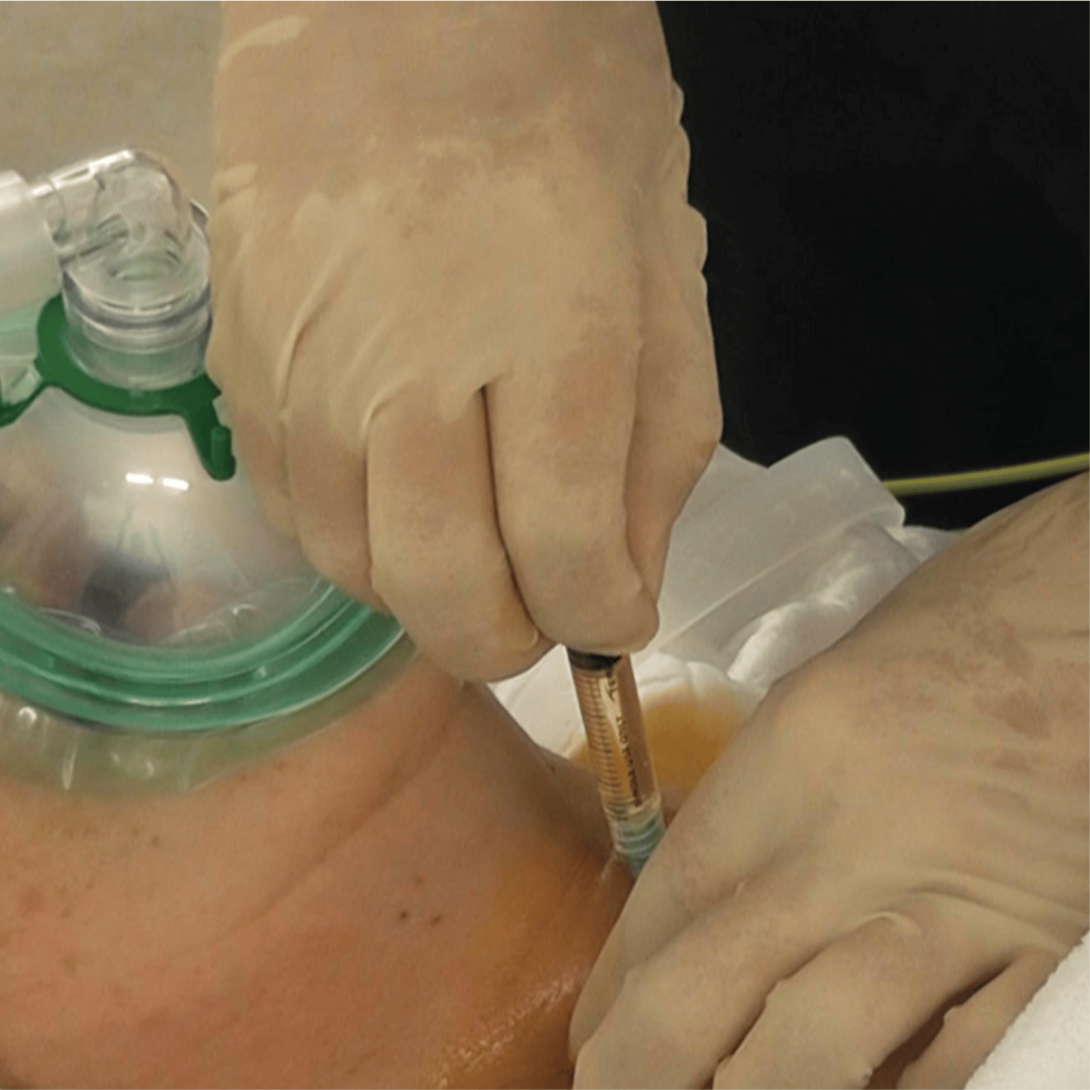
Translaryngeal block targeting the sensory nerves of the trachea below the vocal cords Patients often cough after a translaryngeal block. Therefore, anesthetists and nurses must always wear face shields.

After identifying the greater cornu of the hyoid bone or the superior cornu of the thyroid cartilage, a 25-gauge needle was inserted laterally, and 1 mL of 1% lidocaine was administered bilaterally. For the translaryngeal block, the patient's head was slightly extended, and the thyroid notch, lower thyroid cartilage edge, and cricoid cartilage were palpated (Figure 3). Thereafter, a 23-gauge needle was inserted into the cricothyroid ligament, and 2 mL of 4% lidocaine was injected. Reflex coughing distributed the anesthetic, providing effective surface anesthesia below the vocal cords. If nasal intubation was required, the nasal cavity was disinfected, and bleeding was controlled before performing the nerve block. Additional pethidine or fentanyl was administered as required to manage patient movement during the procedure. After visualizing the vocal cords using a fiberoptic bronchoscope, the tracheal tube was guided into the trachea. Successful intubation was confirmed using capnography, and propofol was subsequently administered.

## Results

A case in which the 3N technique was used for nasal intubation

A 70-year-old male patient with a tumor involving the entire cervical region was anticipated to have difficult intubation. His medical history included hypertension, and thyroid function tests showed no abnormalities. He was scheduled to undergo surgery under general anesthesia for maxillary cancer. Following the administration of midazolam, intubation was performed according to the 3N technique (Video 1). Postoperatively, upon assessing the patient's condition, it was confirmed that he had no recollection of events following midazolam administration and experienced no discomfort. The nerve block effectively suppressed reflexes, allowing both the anesthesiologist and assisting nurses to perform anesthesia induction without stress.

**Video 1:** Tracheal intubation maintaining spontaneous breathing technique with nerve blocks We introduce a new approach that employs these advanced nerve block techniques for difficult airway management.

Retrospective analysis

Clinical records of 18 consecutive patients who underwent anesthesia management using the 3N technique between January 1, 2023, and January 31, 2025, were collected (Table 1). Only cases in which awake intubation was performed at our hospital were included. This study focused on cases typically associated with DAM, such as limited cervical extension, abnormal oral anatomy, and restricted mouth opening. Cases in which communication was not possible after surgery were excluded.

**Table 1:** Patient characteristics The table provides background information on 18 patients who underwent treatment using the 3N technique. "Memory" was assessed post-surgery to determine if the patient retained any memories of the period between sedative administration and intubation. BMI: Body mass index

Case	Gender	Age	Height (cm)	Weight (kg)	BMI	Reasons for Awake Intubation	Intubation
1	Male	75	161	68	26	Cervical spondylotic myelopathy (C4-5)	Nasal
2	Male	76	154	56	24	Difficulty in neck extension due to an anterior cervical tumor	Nasal
3	Female	76	149	68	31	Trismus due to mandibular osteomyelitis	Nasal
4	Female	42	153	131	56	Severe obesity	Oral
5	Male	60	173	68	23	Postoperative laryngeal cancer	Oral
6	Male	18	177	58	19	Trismus due to a zygomatic bone fracture	Oral
7	Male	42	178	77	24	Mandibular hypoplasia	Nasal
8	Male	71	165	53	19	Ossification of the posterior longitudinal ligament (C4-5)	Nasal
9	Male	87	157	63	26	Cervical spinal cord injury	Oral
10	Male	75	160	73	29	Cervical degeneration, cervical instability (C5-7)	Nasal
11	Male	55	184	178	53	Severe obesity	Oral
12	Female	54	146	40	19	Mandibular hypoplasia	Nasal
13	Female	39	161	64	25	Trismus due to temporomandibular joint disorder	Nasal
14	Male	80	170	101	35	Upper airway obstruction due to tongue cancer	Nasal
15	Female	71	160	61	24	Trismus due to postoperative oral cancer	Nasal
16	Female	83	141	60	30	Cervical degeneration, cervical instability (C5-6)	Nasal
17	Female	71	160	60	23	Trismus due to postoperative oral cancer	Nasal
18	Male	60	165	57	21	Trismus due to tongue cancer	Nasal

We collected data on the time from anesthesia initiation to intubation, types and dosages of sedatives and analgesics, patient discomfort during induction (assessed using a visual analog scale), and memory at induction. Among the 18 patients who underwent the 3N technique for the management of severely difficult airways, the indications for ATI included obesity, restricted mouth opening, inability to extend the neck, and anatomical abnormalities of the upper airway. In all cases, postoperative interviews revealed that the patients had no memory of events following sedative administration (Table 2). Consequently, none of the patients experienced nerve block or intubation-related events. The discomfort level, assessed using the visual analog scale, was 0/100 for all patients. The mean time from anesthesia induction to successful intubation was 16 minutes.

**Table 2:** Results of the 3N technique "Time A" and "Time B" were measured in minutes. Discomfort during induction was assessed using a visual analog scale. MDZ: Midazolam; Fen: Fentanyl; Pet: Pethidine; Time A: Duration from operating room entry to intubation; Time B: Duration from sedation initiation to intubation; Dis: Discomfort during induction.

Case	Sedatives	Opioids	Time A	Time B	Memory	Dis
1	MDZ 4 mg	-	17	14	Sedation start	0
2	MDZ 5 mg	Fen 100 μg	20	16	Sedation start	0
3	MDZ 5 mg	-	21	14	Sedation start	0
4	MDZ 10 mg	-	19	18	Sedation start	0
5	MDZ 4 mg	Fen 50 μg	37	15	Sedation start	0
6	MDZ 7.5 mg	Fen 25 μg	17	15	Sedation start	0
7	MDZ 7 mg	Fen 25 μg	17	16	Sedation start	0
8	MDZ 5 mg	Fen 100 μg	20	18	Sedation start	0
9	MDZ 2.5 mg	Fen 50 μg	27	19	Sedation start	0
10	MDZ 6 mg	-	14	13	Sedation start	0
11	MDZ 7 mg	Pet 70 mg	18	11	Sedation start	0
12	MDZ 5 mg	Fen 50 μg	19	17	Sedation start	0
13	MDZ 6 mg	Fen 75 μg	26	23	Sedation start	0
14	MDZ 7 mg	Pet 70 mg	29	17	Sedation start	0
15	MDZ 7 mg	Fen 25 μg	22	18	Sedation start	0
16	MDZ 6 mg	Pet 35 mg	16	14	Sedation start	0
17	MDZ 4 mg	Pet 35 mg	12	11	Sedation start	0
18	MDZ 9 mg	Pet 35 mg	25	24	Sedation start	0
Average	-	-	21	16	-	-
SD	-	-	6	3	-	-

## Discussion

Herein, we propose a novel approach to DAM, characterized by reduced patient burden and decreased stress for anesthesiologists. Mohanta et al. reported significantly lower time requirements for performing awake fiberoptic intubation when patients received ultrasound-guided airway nerve block for airway anesthesia, compared to ultrasonic nebulization [16]. The current study suggests that nerve blocks are effective during awake intubation.

The key features of the 3N technique include (1) reflex suppression during intubation, which facilitates smooth procedural execution; (2) preservation of spontaneous breathing, which eliminates the apnea time, thus allowing for a more controlled approach; (3) minimal patient movement, reducing the need for extensive support staff during anesthesia induction; and (4) absence of specialized equipment apart from an ultrasound device.

Although the 3N technique requires additional time for nerve block administration, its applicability is broad. For instance, the 3N technique was successfully performed in a patient with mild intellectual disability (Case 14). Thus, provided basic communication is possible, the procedure can be safely performed even in patients with disabilities. Additionally, the availability of reversal agents, such as flumazenil and naloxone, allows for the rapid cessation of sedative and analgesic effects, thereby enhancing safety [17]. While some anesthesiologists may avoid ATI because it requires extensive manpower and specialized equipment, the 3N technique has the potential to overcome these limitations. The dosage of sedatives and opioids requires further consideration. Using a bispectral index monitor might have allowed for more precise adjustments. The 3N technique requires the administration of a precise amount of local anesthetic agents. It is crucial to ensure that the total dosage does not exceed the maximum recommended limit, which is typically determined based on the patient's body weight and other individual factors. Exceeding these dosage thresholds significantly increases the risk of local anesthetic systemic toxicity, a potentially life-threatening condition characterized by central nervous system and cardiovascular complications. Therefore, meticulous calculation and strict adherence to established dosing guidelines are essential to mitigate the risk of systemic toxicity associated with local anesthetic administration.

This technique has some limitations. Particularly, the 3N technique is unsuitable for patients with a full stomach, and its use in pediatric cases remains untested. For patients with contraindications to midazolam, alternative sedatives may be viable options, such as dexmedetomidine or remimazolam [18-20]. Moreover, although pethidine causes minimal respiratory depression among opioids, continuous monitoring of the respiratory status remains critical.

Compared with conventional methods, the 3N technique can be applied to several situations in "cannot ventilate, cannot intubate" scenarios. This simple, low-risk technique may also be applicable to patients with DAM. Although further studies are needed, our novel 3N technique could serve as a simple alternative to conventional methods in routine clinical practice. In the future, it may be considered one of the less invasive approaches recommended in the ATI guidelines.

## Conclusions

The novel 3N technique for DAM suppresses the gag and glottic closure reflexes through nerve blocks. These effects may facilitate intubation for anesthesiologists who have previously avoided ATI. Moreover, this technique is a less stressful approach for both anesthesiologists and patients, as it minimizes discomfort.
